# Biological Aging, Immune Phenotypes, and Susceptibility to COVID-19 and Sepsis: A Mendelian Randomization Study

**DOI:** 10.1016/j.virusres.2026.199756

**Published:** 2026-06-02

**Authors:** Yuru Tang, Xiao Gao, Huafang Ding, Lingli Kong, Xiaoyan Zhu

**Affiliations:** a Department of Anesthesiology, Qingdao Municipal Hospital, Qingdao, Shandong, China;; b Department of Geriatrics, Qingdao Mental Health Center, Qingdao University, Qingdao, Shandong Province, China.; c Department of Critical Care Medicine, The Affiliated Hospital of Qingdao University, Qingdao, Shandong, China;

**Keywords:** Biological Aging, COVID-19, Sepsis, Immune Cells, Lifestyles

## Abstract

Aging is a biological process characterized by time-dependent cellular and functional decline, leading to progressive deterioration of physiological integrity. Growing evidence suggests that biological aging is accompanied by increased morbidity and mortality of COVID-19. Biological aging may serve as biomarkers for assessing immune health and improving risk stratification. The underlying genetic phenotypes of aging are complex. We used multivariate genome-wide association data with multidimensional aging traits 'MV-Age' and telomere length (TL) to assess genetically informed associations between biological aging signatures and COVID-19 as well as sepsis. To inform potential public health strategies, we also performed Mendelian randomization (MR) analysis to explore whether biological aging may mediate the associations of modifiable lifestyle and infectious disease. We next examined biological aging-associated immunocompetence in relation to COVID-19 and sepsis. Our findings provide a detailed landscape of immune-senescence with genetic association to biological aging and COVID-19 as well as sepsis, facilitating the development of advanced biological aging signature, assessing interventions, and studying reversibility of age-associated infectious changes. Our study further found specific immunocyte phenotypes that could significantly improve clinical outcomes in COVID-19 and sepsis through their relationship with healthy biological aging.

## Highlights

• Demonstrate causal evidence that biological aging and aging-associated immune cell types represent independent risk factors for COVID-19 and sepsis.
• Explore the role of age-related immune changes as a potential mechanism underlying increased infection vulnerability, thereby informing targeted strategies for geriatric populations.
• Assess the mediating effects of modifiable aging-related lifestyles (specifically healthy weight and smoking behaviors), highlighting actionable targets to reduce infectious disease burden in older individuals.

## Graphical abstract

**Figure:**
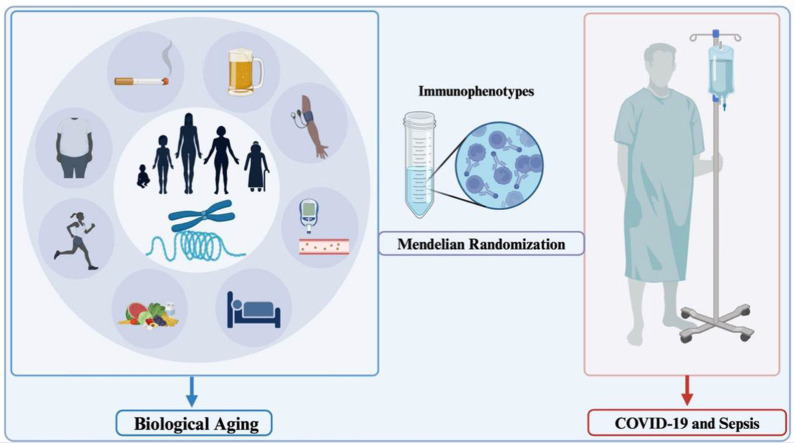

**Abbreviation**
BFP: Body fat percentage
BMI: Body mass index
CI: Confidence interval
DBP: Diastolic blood pressure
GWAS: Genome-wide association studies
GLGC: Global Lipids Genetics Consortium
GSCAN: GWAS and Sequencing Consortium of Alcohol and Nicotine use
HbA1c: Hemoglobin A1c
HDL: High-density lipoprotein
HGI: Host Genetics Initiative
ICBP: International Consortium of Blood Pressure
IVW: Inverse-variance weighted
LDL: Low-density lipoprotein
MR: Mendelian randomization
MR-PRESSO: Mendelian randomization pleiotropy residual sum and outlier
MVMR: Multivariate MR
OR: Odds ratio
SNP: Single nucleotide polymorphism
SSGAC: Social Science Genetic Association Consortium
TL: Telomere length
TC: Total cholesterol
TG: Triglyceride
UVMR: Univariate MR
WC: Waist Circumference
WFGC: Within Family GWAS Consortium

## 1 Introduction

The average human life expectancy has risen markedly worldwide since the beginning of the 21^st^ century(Roth et al., 2018; Partridge et al., 2018). Extended aging has not been shaped by natural selection, which makes older populations vulnerable to a variety of chronic and degenerative diseases, reduces their quality of life, and increases the burden of healthcare costs(Mavromatis et al., 2023; Partridge et al., 2018). Life-threatening infectious conditions are responsible for a substantial impact on the global healthcare burden; thus, primary prevention strategies are of utmost importance(Komorowski, 2020; Rudd et al., 2020; Stanski and Wong, 2020). Since the coronavirus disease 2019 outbreak, the role of biological aging has been proposed as a critical risk factor influencing the severity of infectious disease(Liu et al., 2023; Tian et al., 2023). Aging associated decline in intrinsic capacity makes increased morbidity and mortality causes of infectious disease in elderly individuals(Schmitt et al., 2023). Infectious diseases in the elderly group are significantly high worldwide, which are more likely to develop new functional impairments compared with younger individuals(Manabe and Heneka, 2022; Singer et al., 2019). Potential reversibility of biological aging signature, effectively modifiable risk factors tailored approach for infectious disease and their consequences, is likely to have the greatest impact on morbidity and improving overall quality of life(Cohen et al., 2015; Lee et al., 2022; Trinder et al., 2020).

Recent evidence has gradually recognized that short-term treatments of the immune system might have long-term effects, keeping side effects to a more manageable minimum, particularly anti-infectious therapy in terms of infectious progression(Gorgoulis et al., 2019; Abbott, 2024). Indeed, adults with chronic inflammatory conditions have a heightened risk for developing severe COVID-19 and dying(Huang et al., 2020). Given the clearly greater susceptibility of older adults to COVID-19, the interconnection between immunity and senescence is now receiving unprecedented urgency during the SARS-CoV-2 pandemic, bringing to the fore the critical need to improve older people's immune function and resilience(Pawelec et al., 2020). Recent years have seen growing exploration of the genetic architecture of sepsis, a highly heterogeneous clinical syndrome characterized by dysregulated host responses to infection(Tong et al., 2025; Wang et al., 2024). Infectious diseases may have an exacerbated clinical course in older people due to a virulent immune response, increased susceptibility of immune cells to immune-mediated damage, or a combination of these factors(Zinatizadeh et al., 2023). As decline and deficits of immune system contributes to infection-related disease in humans, in this regard, making immunophenotypes may be one of exciting targets for optimal pro-longevity intervention at the systemic level.

Enormous progress has identified measurements at multiple landscape of biological aging contribute to modulating health aging and lifespan(Cohen et al., 2022). Aging-related modifiable behavior risk factors including smoking behaviors, healthy dietary habits, regular physical exercise, alcohol consumption, obesity, and metabolic health indices have all been reported(Bountziouka et al., 2022; Lloyd-Jones et al., 2022; Oblak et al., 2021; The China Kadoorie Biobank Collaborative Group, 2023). Observational studies have suggested that modifiable lifestyles have the potential role in enhancing healthy longevity and preventing the morbidity and mortality of infectious disease(Orkaby, 2021; Paulsen et al., 2017). While it is reported the susceptibility of symptoms of infectious disease is strongly associated with aging, little is known about whether biological aging-related status and what extent aging-related healthy lifestyles influence risk and mortality of COVID-19 and sepsis.

Recent Mendelian randomization (MR) analyses provide important information for epidemiologists, clinicians, and public health practitioners about the genetic association of disease outcomes(Emdin et al., 2017; Russell et al., 2022; Sanderson et al., 2022). MR is a causal inference approach that leverages naturally occurring genetic variants as instrumental variables to minimize confounding and assess the genetic association between a biological trait and disease risk(Yao et al., 2022). Over the past decade, genome-wide association studies (GWAS) involving millions of genetic variants across the genomes of many individuals have achieved clear success in identifying and translating novel disease susceptibility genes into clinical interventions(Grotzinger et al., 2019; Tam et al., 2019). Aging is a multifaceted process shaped by genetic, epigenetic, and environmental influences. Multiple strategies have been developed to quantify biological aging, including telomere dynamics, physiological aging indicators, and DNA methylation–based epigenetic clocks such as Horvath, Hannum, and GrimAge(Argentieri et al., 2025; Atkins et al., 2021; Kroemer et al., 2025; Ying et al., 2024).

To explore the inherited component of aging and its relevance to infection susceptibility, we focus on our analysis on SNP-based pathways that genetically connect age-related biology with disease risk. Using multidimensional aging-related summary-level GWAS datasets, our study focuses on the heritable component of aging by applying SNP-based biological aging measures as instruments to evaluate the potential associations on susceptibility to COVID-19 and sepsis. To further explore the genetically predicted associations between biological aging and infectious outcomes, we conducted exploratory analyses to evaluate whether immunocyte phenotypes may represent potential intermediates linking aging-related traits to COVID-19 and sepsis. We additionally performed MR analyses to examine the genetically predicted associations between aging-related modifiable lifestyle factors and COVID-19 as well as sepsis, which may provide insights relevant to public health strategies for healthy aging.

## 2 Methods

### 2.1 Study Design

**Figure 1:**
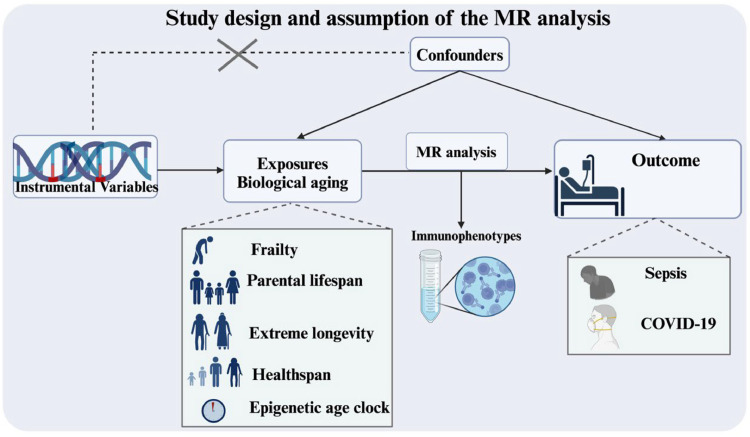
Graphical depiction of the framework and methods employed in the Mendelian randomization study.

We employed a comprehensive three-phase study design to evaluate the potential clinical implications of our findings (Figure 1). MR analyses were firstly performed to examine the presence of bidirectionality between biological aging phenotypes and risk of COVID-19 as well as sepsis. In addition, we used univariate MR (UVMR) and multivariate MR (MVMR) to examine the relationships of aging-related modifiable lifestyles and sepsis outcomes. Finally, the lifestyle factors identified as associated with sepsis in the multivariable MR analysis were further examined in a COVID-19 dataset to explore the consistency of these associations across related infectious outcomes(Burgess and Thompson, 2015; Sanderson et al., 2018). To explore the potential relationship between biological aging and infectious diseases, we further conducted mediational MR analysis to investigate whether specific 731 immunocyte phenotypes as mediators of biological aging and infectious diseases(Orrù et al., 2020). Then, we implemented mediated MR analysis to assess whether the associations between selected modifiable lifestyle factors and infectious diseases risk were mediated by biological multi-aging phenotypes(Carter et al., 2021).

### 2.2 Data Sources

We included 472,174 participants who underwent leukocyte telomere length (TL) measurement(Codd et al., 2021), and the genetic associations with the summary level of multi-biological aging indices were derived from a large GWAS summary meta-analysis data that included 1,958,774 European participants(Rosoff et al., 2023). Multi-aging-related traits including health life expectancy, parents lifespan(Timmers et al., 2019), extreme human longevity(Deelen et al., 2019), frailty(Atkins et al., 2021) and epigenetic clock(McCartney et al., 2021) (Pheno-Age, Grim-Age, Intrinsic Horvath-Age, and Hannum-Age). We used summary statistics information of infectious diseases from a centralized meta-analysis data COVID-19 (32,519 cases and 2,062,805 controls), sepsis (11,643 cases and 474,841 controls). We identified 30 potentially aging-related lifestyle candidates that could potentially influence sepsis after reviewing previously reported literature(Lloyd-Jones et al., 2022; Ponsford et al., 2020). These 30 potentially modifiable essential lifestyle factors were organized into 8 major categories: cigarette smoking (four traits), moderate alcohol drinking (three traits), healthy diet (four traits), physical inactivity (four traits), healthy sleep (three traits), maintenance of a healthy body weight (three traits), anthropometric metabolic index (four lipid and three glucose traits), and blood pressure (two traits). The GWAS catalog (GCST90001391 to GCST90002121) provides an overview of GWAS statistics for 731 immunophenotypes collected from 3757 Sardinians, a specific European subgroup(Orrù et al., 2020). All data were obtained from reliable consortia or studies based on summary-level statistics from GWASs or GWAS meta-analyses conducted in individuals of European descent. Current information on the GWAS datasets utilized in this study is presented in Table 1.

**Table 1:** Characteristics of data in this study.

Traits	Consortium	Participants (individuals)	n. SNP	Web address where data is available
Biological aging				
TL	NA	472,174	20,134,421	https://opengwas.io/datasets/ieu-b-4879
MV-age	GWAS meta	1,958,774	6793878	https://zenodo.org/record/7926323
Smoking				
Smoking Initiation	GSCAN	3,383,199	13,595,219	https://conservancy.umn.edu/handle/11299/241912
Smoking Cessation	GSCAN	3,383,199	13,642,427	https://conservancy.umn.edu/handle/11299/241912
Cigarettes per day	GSCAN	3,383,199	13,763,312	https://conservancy.umn.edu/handle/11299/241912
Smoking status: Never	Neale Lab	359,706	13,586,591	https://gwas.mrcieu.ac.uk/datasets/ukb-d-20116_0/
Alcohol intake				
Drinks Per Week	GSCAN	3,383,199	13,268,540	https://conservancy.umn.edu/handle/11299/241912
Alcohol intake versus 10 years previously	Neale Lab	313,248	10,894,596	https://opengwas.io/datasets/ukb-a-32
Alcohol intake frequency	Neale Lab	336,965	10,894,596	https://opengwas.io/datasets/ukb-a-25
Dietary intake				
Protein	SSGAC	268,922	1141,7548	https://www.thessgac.org/
Sugar	SSGAC	235,391	11417548	https://www.thessgac.org/
Carbohydrate	SSGAC	268,922	11417548	https://www.thessgac.org/
Fat	SSGAC	268,922	11417548	https://www.thessgac.org/
Physical activity				
Leisure screen time	GWAS meta	703,901	20,320,957	https://www.ebi.ac.uk/gwas/studies/GCST90104339
Sedentary behavior at work	GWAS meta	703,901	47,070,757	https://www.ebi.ac.uk/gwas/efotraits/EFO_0008002
Moderate to vigorous physical activity levels	GWAS meta	703,901	22,586,718	https://www.ebi.ac.uk/gwas/efotraits/EFO_0008002
Vigorous physical activity	NA	261,055	11,803,978	https://opengwas.io/datasets/ebi-a-GCST006098
Sleep health				
Getting up	GWAS meta	1,331,010	10862567	https://ctg.cncr.nl/software/summary_statistics
Sleep duration	GWAS meta	1,331,010	10862567	https://ctg.cncr.nl/software/summary_statistics
Morningness	GWAS meta	1,331,010	10862567	https://ctg.cncr.nl/software/summary_statistics
Blood pressure				
Diastolic blood pressure	ICBP	757,601	7,160,619	https://opengwas.io/datasets/ebi-a-GCST90025981
Systolic blood pressure	ICBP	757,601	7,088,083	https://opengwas.io/datasets/ebi-a-GCST90025968
Metabolic factor				
HDL	WFGC	37120	7892377	https://opengwas.io/datasets/ieu-b-4843
Log TG	GLGC	1,654,960	47196261	http://csg.sph.umich.edu/willer/public/glgclipids2021/
TC	GLGC	1,654,960	46513217	http://csg.sph.umich.edu/willer/public/glgclipids2021/
LDL	GLGC	1,654,960	47006483	http://csg.sph.umich.edu/willer/public/glgclipids2021/
Fasting insulin	NA	151,013	29,664,438	https://opengwas.io/datasets/ebi-a-GCST90002239
Fasting glucose	NA	200,622	31,008,728	https://opengwas.io/datasets/ebi-a-GCST90002232
HbA1c	WFGC	45,734	9,696,819	https://opengwas.io/datasets/ieu-b-103
Healthy bodyweight				
WC	Neale Lab	336639	10,894,596	https://opengwas.io/datasets/ieu-a-61
BMI	Neale Lab	336,107	10,894,596	https://opengwas.io/datasets/ukb-a-248
BFP	Neale Lab	331,117	10,894,596	https://opengwas.io/datasets/ukb-a-264
Infectious diseases				
Sepsis	UK Biobank	486,484	12,243,539	https://opengwas.io/datasets/ieu-b-4980
COVID-19	HGI	2,095,324	12,484,091	https://www.covid19hg.org/results/r7/
Immunophenotypes	Sardinians	3757	18,238,300	http://ftp.ebi.ac.uk/pub/databases/gwas/summary_statistics/

Body fat percentage (BFP); Body mass index (BMI); Diastolic blood pressure (DBP); Global Lipids Genetics Consortium (GLGC); GWAS and Sequencing Consortium of Alcohol and Nicotine use (GSCAN); Hemoglobin A1c (HbA1c); High-density lipoprotein (HDL); Host Genetics Initiative (HGI); International Consortium of Blood Pressure (ICBP); Low-density lipoprotein(LDL); Number of SNPs (n. SNP); Single nucleotide polymorphism (SNP); Social Science Genetic Association Consortium (SSGAC); Telomere length(TL); Total cholesterol(TC); Triglyceride (TG); WC (waist circumference); Within Family GWAS Consortium (WFGC).

### 2.3 Basic Characteristics of the MR Study

This study followed the guidelines of the Strengthening the Reporting of Observational Studies in Epidemiology MR statement report (Skrivankova et al., 2021). To support the validity of MR-based inferences, three core assumptions should be considered. First, a strong and vigorous association exists between genetic instrumental variables (IVs) and the exposure of interest. Second, the genetic IVs should not be associated with any potential confounders between each potential exposure and the outcome. Third, the effects of genetic IVs influence the outcomes only through the exposures of interest, without any involvement in direct or indirect pathways (Emdin et al., 2017; Hemani et al., 2018). In this study, single nucleotide polymorphisms (SNPs) were used as risk factors in instrumental variable genetic analysis based on summary-level data. For each exposure such as telomere length and MV-aging, genome-wide significant SNPs identified from large-scale GWAS were selected as instrumental variables. After harmonizing effect alleles between the exposure and outcome datasets, MR analyses were conducted to estimate the associations of the exposures on the outcomes. To select independent genetic variants and verify the robustness of the main findings, a stringent condition excluded all genetic variants that were genome-wide significant (*P*<5*10^−8^) with a linkage disequilibrium threshold of R^2^<0.001, a window of 10,000 kb, which was restricted to minimize potential bias arising from linkage disequilibrium. The number of SNPs retained for each trait is reported in the Table 2 and Supplementary Tables.

**Table 2:** UVMR estimates as well as the pleiotropy test and heterogeneity test for the enetically predicted associations of each biological aging (TL and MV-Age) with COVID-19 and Sepsis.

**Exposure**	**Outcome**	**Method**	**n. SNP**	**β**	**SE**	***P*-_value_**	**OR (95%CI)**
**TL**	**Sepsis**	**MR Egger**	141	0.205	0.093	0.029	1.228(1.024, 1.472)
		**Weighted median**		0.099	0.085	0.242	1.105(0.935,1.305)
		**IVW**		0.137	0.052	0.009	1.146(1.035, 1.270)
		**Simple mode**		0.250	0.196	0.204	1.284(0.874,1.886)
		**Weighted mode**		0.228	0.098	0.021	1.256 (1.037, 1.522)
**MV-Age**	**Sepsis**	**MR Egger**	32	-0.996	1.000	0.327	0.369(0.052, 2.619)
		**Weighted median**		-0.878	0.494	0.075	0.416 (0.158, 1.094)
		**IVW**		-1.160	0.379	0.002	0.314(0.149, 0.659)
		**Simple mode**		-1.703	1.061	0.119	0.182(0.023, 1.458)
		**Weighted mode**		-1.049	0.785	0.191	0.350(0.075, 1.633)
**TL**	**COVID-19**	**MR Egger**	120	0.079	0.072	0.279	1.082(0.939, 1.246)
		**Weighted median**		0.061	0.072	0.3912	1.063(0.924,1.224)
		**IVW**		0.096	0.041	0.019	1.101(1.016, 1.194)
		**Simple mode**		0.117	0.134	0.387	1.124(0.864,1.462)
		**Weighted mode**		0.033	0.066	0.616	1.034(0.908, 1.176)
**MV-Age**	**COVID-19**	**MR Egger**	30	0.419	0.748	0.580	1.521(0.351, 6.592)
		**Weighted median**		0.841	0.383	0.028	2.319 (1.095, 4.910)
		**IVW**		0.828	0.272	0.002	2.289(1.343, 3.900)
		**Simple mode**		1.549	0.790	0.060	4.705(0.999, 22.147)
		**Weighted mode**		1.450	0.752	0.064	4.263(0.975, 18.626)

Confidence interval (CI); Inverse-variance weighted (IVW); Mendelian randomization (MR); Mendelian randomization pleiotropy residual sum and outlier (MR-PRESSO); Number of SNPs (n. SNP); Odds ratio (OR); Standard error (SE); Telomere length (TL); Univariable Mendelian randomization (UVMR).

### 2.4 Colocalization analyses

Colocalization analyses were performed using the coloc framework to assess whether the same genetic variants are likely responsible for both exposures (BMI, MV-Age, TL) and outcomes (COVID-19, sepsis), with posterior probability PP_4_≥0.8 considered supportive of shared causal variants, and were used to complement MR analyses.

### 2.5 Statistical Analyses

The MR analyses were predominantly conducted using the inverse-variance weighted (IVW) method to determine the odds ratio (OR) and 95% confidence interval (CI) for the relationship association of each modifiable lifestyle and COVID-19 as well as sepsis(Bowden et al., 2017). To reduce the potential impact of weak instruments, we evaluated instrument strength using the F-statistic and retained only variants with F>10(Burgess et al., 2011). The IVW method provides unbiased causal estimates under the core assumptions of MR, provided that the instrumental variables (IVs) are valid. Because of the possibility of pleiotropic instrumental variables biasing the IVW estimates, additional sensitivity analyses were performed using the MR Egger, MR weighted median, the simple median and weighted mode assessment approaches to offer robustness of results (Bowden et al., 2017, 2016, 2015; Hartwig et al., 2017). When the intercept value differed from zero, MR-Egger regression was used to assess and adjust for potential directional pleiotropy (Bowden et al., 2015; Rees et al., 2017). The Mendelian randomization pleiotropy residual sum and outlier (MR-PRESSO) test was used to identify. Leave-one-out permutation analysis was conducted across all methods to assess the influence of potentially pleiotropic SNPs on the causal estimates (Verbanck et al., 2018). Cochran's Q statistic was used to test heterogeneity, a *P*_value_ of less than 0.05 were considered significant heterogeneity (Corbin et al., 2016). The Radial plot can detect the outlying variants and influential data points(Bowden et al., 2018). To reduce potential bias arising from sample overlap in the GWAS datasets, we applied strong instrumental variables and conducted multiple sensitivity analyses, including MR-Egger, weighted median/mode, MR-PRESSO, and leave-one-out tests. Once pleiotropy and heterogeneity were present, potential outlier SNPs influencing the results were eliminated using MRPRESSO(Verbanck et al., 2018), leave-one-out methods(Corbin et al., 2016) or radial plot variants of the IVW(Bowden et al., 2018). After removing IVs with pleiotropic or effects heterogeneity, we repeated the main MR analysis. Bonferroni-correction of the significance threshold with *P*_value_<0.05/(number of traits under study) as the significance level(Curtin and Schulz, 1998). We applied the Benjamini–Hochberg false discovery rate (FDR) method to identify possible aging-related immunophenotypes while controlling the expected number of false positives (EPIC-CVD Consortium et al., 2017). A ‘product of coefficients’ method was utilized to assess the indirect effect on COVID-19 and sepsis risk via each potential mediator. All MR analyses were performed using R software (version 4.2.2) using R Studio (version 2024.12.1.563). Additional R packages, including TwoSample MR, MRPRESSO, Radial MR and ggplot2 were used for data processing and visualization. Detailed descriptions of all software tools and analytical parameters used in each MR analysis are provided in the Supplementary Figures.

## 3 Results

### 3.1 Genetic Associations between Genetically Predicted Biological Aging and the Risk of COVID-19 and Sepsis

We applied a two-sample UVMR approach to evaluate the association of each genetic instrument of biological aging phenotypes on the risk of infectious diseases. We observed significant genetically associations between TL and sepsis outcomes (OR_IVW_:1.164; 95% CI_IVW_, 1.035 to 1.270; *P*_IVW_=0.009) as well as between genetically predicted MV-Age indices and sepsis outcomes (OR_IVW_:0.314; 95% CI_IVW_, 0.140 to 0.659; *P*_IVW_=0.002) (Table 2). Meanwhile, we found statistically significant genetic evidence for linking the effects of TL (OR_IVW_:1.101; 95% CI_IVW_, 1.016 to 1.094; *P*_IVW_=0.019) and MV-age indices (OR_IVW_:2.289; 95% CI_IVW_, 1.343 to 3.900; *P*_IVW_=0.002) on COVID-19 outcomes (Table 2 and Supplementary Figure 17-18).

To investigate whether these associations reflect shared genetic architecture, we conducted colocalization analyses. MV-Age and COVID-19 exhibited moderate support for shared causal variants (PP.H_4_=0.817), whereas telomere length (TL) and COVID-19 showed low support (PP.H_4_=0.021). For sepsis, colocalization analyses revealed minimal evidence of shared causal variants with MV-Age (PP.H_4_= 0.005) and similarly low support with TL (PP.H_4_=0.054). Scatter plots, forest plots, 'leave-one-out', radial plots, and funnel plots were used to visualize the genetically predicted associations and to identify potential outliers, influential variants, and directional pleiotropy (Supplementary Figures 13-14). After removing the identified outlier SNPs, we reassessed pleiotropy and heterogeneity and found no significant evidence of horizontal pleiotropy or heterogeneity for either TL or MV-Age in sepsis or COVID-19 analyses, as shown by the non-significant Cochran's Q-statistics (Supplementary Tables 4-5).

### 3.2 Associations of Aging-Related Modifiable Lifestyles with the Risk of COVID-19 and Sepsis

**Figure 2:**
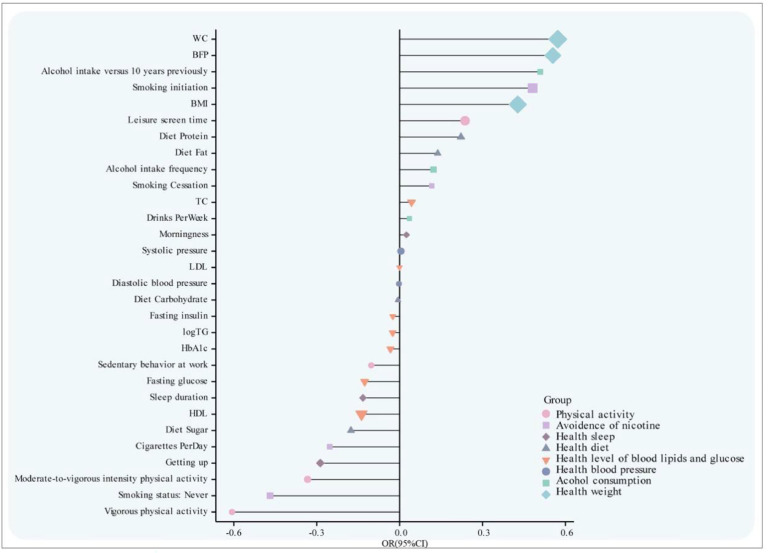
**Lollipop graph representation of the associations of aging-related modifiable lifestyle factors with sepsis risk.** Lollipop plot showing the percentage of 8 essential modifiable lifestyle factors with the results of the IVW (inverse-variance weighted) test. Body fat percentage (BFP); Body mass index (BMI); Diastolic blood pressure (DBP); Hemoglobin A1c (HbA1c); High-density lipoprotein (HDL); Low-density lipoprotein (LDL); WC (waist circumference); Triglyceride (TG); Total cholesterol (TC).

**Figure 3:**
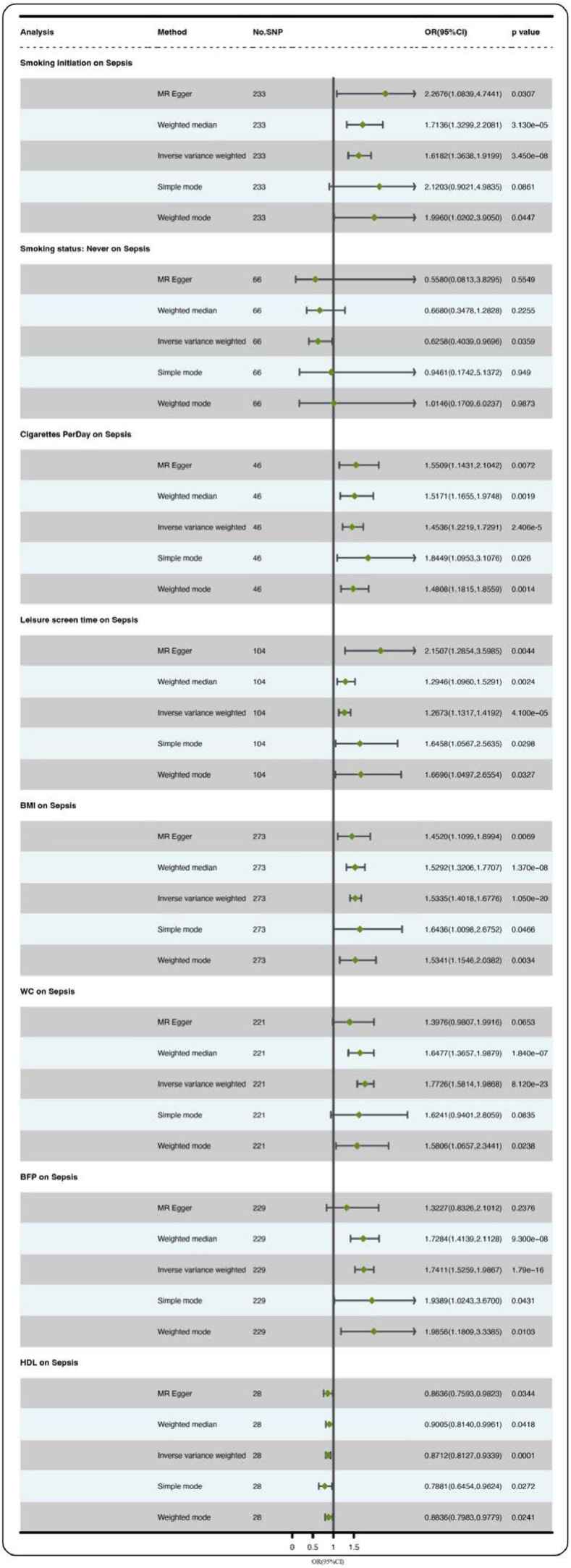
**UVMR estimates of the genetically predicted associations of the eight essential modifiable lifestyle factors with sepsis outcome.** Diamond patterns represent OR (odds ratio); horizontal bars depict a 95% confidence interval (95%CI). The pink diamond patterns represent the IVW of the UVMR results, purple diamond patterns represent the MR-Egger UVMR results, green diamond patterns with light patterns represent the inverse variance weighted of the UVMR results, yellow diamond patterns represent the simple mode of the UVMR results, and darker green patterns represent the inverse variance weighted of the UVMR results. Body fat percentage (BFP); Body mass index (BMI); High-density lipoprotein (HDL); Univariate Mendelian randomization (UVMR); WC (waist circumference).

After the exposure selection process, eight age-specific modifiable risk factor interventions showed a significant association with sepsis outcomes. Genetically predicted increased levels of anthropometric adiposity indicators (BMI, WC, and BFP), smoking index (smoking initiation and cigarettes per day), and physical inactivity (leisure screen time) were significantly associated with an elevated risk of sepsis. In IVW analyses, genetically predicted higher WC showed the strongest association with sepsis (OR_IVW_: 1.773; 95%CI_IVW_, 1.581 to 1.987; *P*_IVW_=8.12 × 10^−23^); followed by BFP (OR_IVW_: 1.741; 95%CI_IVW_, 1.526 to 1.987; *P*_IVW_=1.79 × 10^−16^); smoking behavior (smoking initiation)(OR_IVW_: 1.618; 95%CI_IVW_, 1.364 to 1.920; *P*_IVW_=3.45 × 10^−8^); BMI (OR_IVW_:1.533; 95%CI_IVW_, 1.402 to 1.678; *P*_IVW_=1.05 × 10^−20^) and leisure sedentary behaviors (leisure screen time)(OR_IVW_:1.267; 95%CI_IVW_, 1.132 to 1.419; *P*_IVW_=4.10 × 10^−5^) (Figure 2, Figure 3). By contrast, the metabolic index lipid HDL (OR_IVW_: 0.871; 95%CI_IVW_, 0.813 to 0.934; *P*_IVW_=1.012 × 10^−4^) and the state of never smoking (OR_IVW_: 0.626; 95%CI_IVW_, 0.404 to 0.970; *P*_IVW_=0.035) were inversely associated with sepsis (Figure 2, Figure 3). Figure 2 presents the full univariable MR results for all lifestyle factors respectively, while Figure 3 highlights those with significant associations.

Colocalization analyses revealed varying support for shared genetic signals between key exposures and infectious disease outcomes. BMI also demonstrated high colocalization with sepsis (PP.H4=0.986) and COVID-19 (PP.H4=0.990), indicating strong shared genetic architecture for these traits. Smoking initiation and sepsis exhibited a high posterior probability for a shared causal variant (PP.H4=0.943), whereas the overlap between smoking initiation and COVID-19 was minimal (PP.H4 = 0.017). Scatter plots, forest plots, 'leave-one-out' analysis, funnel plots, and radial plots were used to illustrate the potential relationship between modifiable lifestyle factors and sepsis risk and to identify potential outlier or influential variants (Supplementary Figure 1-8). After excluding the outlier SNPs, no significant horizontal pleiotropy or heterogeneity was observed for any lifestyle exposure in relation to sepsis, as indicated by the non-significant Cochran's Q statistics in Supplementary Table 1. The sensitivity analyses, including MR Egger, weighted median, and leave one out analyses, showed results consistent with the IVW estimates, supporting the robustness of our findings (Supplementary Table 1).

**Figure 4:**
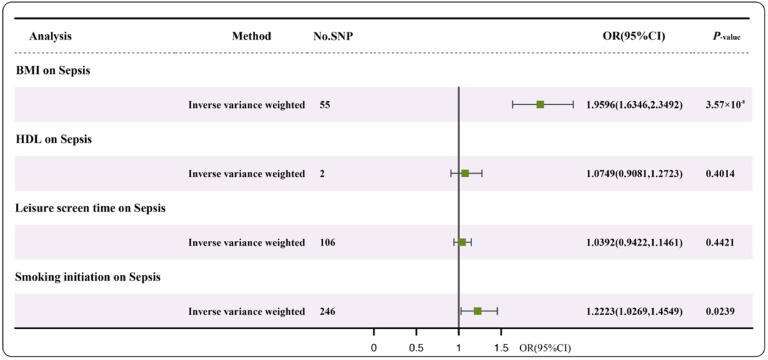
**Forest plot of lifestyle factors with significant positive genetically predicted associations with sepsis risk under the multivariate Mendelian randomization-inverse variance weighted (MVMR-IVW) analysis.** Diamond patterns represent OR (odds ratio); horizontal bars depict a 95% confidence interval (95%CI). The pink diamond patterns represent the IVW of the MVMR results. Body fat percentage (BFP); Body mass index (BMI); High-density lipoprotein (HDL).

To address confounding among modifiable lifestyle factors, we adjusted the MVMR analyses by including the genetic instruments for smoking initiation, smoking status: Never, cigarettes Per Day, leisure screen time, BMI, WC, BFP and HDL (Supplementary Table 2). These instruments acted as trait-specific genetic determinants, allowing us to isolate the independent contribution of each lifestyle exposure. After adjusting for multiple comparisons, smoking initiation and BMI remained statistically significant with sepsis outcomes in the MVMR analysis (Figure 4). BMI exhibited the greatest effect on increased sepsis (OR_IVW_:1.960; 95%CI_IVW_, 1.635 to 2.349; *P*_IVW_=3.569 × 10^−13^) and followed by smoking initiation (OR_IVW_:1.222; 95%CI_IVW_, 1.027 to 1.455; *P*_IVW_=0.024), which were comparable to the univariable IVW estimate. Whereas the magnitude of the associations with leisure sedentary behaviors (OR_IVW_:1.039; 95% CI_IVW_, 0.942 to 1.146; *P*_IVW_=0.442) and HDL (OR_IVW_:1.075; 95% CI_IVW_, 0.908 to 1.272; *P*_IVW_=0.401) with sepsis outcome became no longer statistically significant for sepsis outcome after the multivariate MR. Similarly, the directional consistency and statistical significance of the IVW results in the MVMR analysis were largely in line with those obtained from the MVMR Egger sensitivity analysis, which provides suggestive evidence for a low risk of bias stemming from horizontal pleiotropy (*P*=0.534).

Finally, the lifestyle factors identified as having significant genetically predicted associations with sepsis in the multivariable MR analysis were validated in a COVID-19 data cohort, aiming to examine the consistency of their effects across different related infectious outcomes. BMI exhibited a higher risk of the incident of COVID-19 (OR_IVW_:0.708; 95%CI_IVW_, 0.664 to 0.765; *P*_IVW_= 4.105 × 10^-25^); and smoking initiation was also associated with a statistically significant increase in COVID-19 incidence (OR_IVW_:0.720; 95%CI_IVW_, 0.634 to 0.812; *P*_IVW_=3.299 × 10^-7^).

### 3.3 Mediation Analysis of Biological Aging between Modifiable Essential Lifestyle Genetic Interventions and COVID-19 as Well as Sepsis

Considering the critical role of healthy lifestyle factors in the prevention of aging, biological aging may serve as potential mediators of the protective effect of healthy factors on sepsis outcomes. Firstly, we identified a genetic evidence between BMI (OR_IVW_:0.947; 95% CI_IVW_, 0.928 to 0.966; *P*_IVW_=9.149 × 10^-8^) and smoking initiation (OR_IVW_:0.904; 95% CI_IVW_, 0.881 to 0.927; *P*_IVW_=7.901 × 10^–15^) was associated with TL, respectively(Supplementary Figure 9 and 11). The associations between BMI (OR_IVW_:0.879; 95% CI_IVW_, 0.841 to 0.918; *P*_IVW_=2.716 × 10^-8^), smoking initiation (OR_IVW_: 0.891; 95% CI_IVW_, 0.881 to 0.901; *P*_IVW_=9.994 × 10^–91^) and MV-Age traits were generally statistically significantly, respectively(Supplementary Figure 10 and 12).

Colocalization analyses revealed strong support for shared genetic signals between key exposures and infectious disease outcomes. Smoking initiation and telomere length (TL) showed very strong colocalization (PP.H4=0.995), while smoking initiation and MV-Age displayed moderate support (PP.H4=0.333). BMI and MV-Age showed strong shared genetic variants (PP.H4=0.988), whereas BMI and TL had weaker evidence (PP.H4=0.167).

Next, we conducted a mediator MR analysis to determine the potential mediating pathways between genetically predicted modifiable essential lifestyle factors (BMI and smoking initiation) and the risk of sepsis mediated through biological aging straits (Table 3). We observed that TL and MV-Age may partially account for the association between BMI and sepsis, with estimated indirect proportions of 1.586% (95% CI_IVW_, −0.013 to −0.001) and 31.467% (95% CI_IVW_, 0.039 to 0.223), respectively. The mediating proportion of TL for the genetically predicted association between smoking initiation and sepsis outcome was 2.589% (95% CI_IVW_, -0.024 to -0.003), while MV-age for the genetically predicted association between smoking initiation and sepsis outcome was 23.467% (95% CI_IVW_, 0.011 to 0.019). We then examined whether TL may contribute to the associations between BMI and smoking initiation with COVID-19, with estimated indirect proportions of 2.633% (95% CI_IVW_, −0.017 to −0.003) and 2.057% (95% CI_IVW_, -0.030 to -0.006), respectively. The mediating proportion of MV-age for the genetically predicted association between BMI and smoking initiation with COVID-19 outcome was 11.286% (95% CI_IVW_, -0.156 to -0.008) and 12.125% (95% CI_IVW_, -0.167 to -0.036) respectively.

**Table 3:** The mediation effect of biological aging on the pathway of BMI and smoking initiation with infectious disease.

Exposure	Mediator	Outcome	Direct effect (A)OR (95% CI)	Direct effect (A)β	Direct effect (A)SE	Direct effect (B)OR (95% CI)	Direct effect (B)β	Direct effect (B)SE	Total effect (C)OR (95% CI)	Total effect (C)β	Mediation effect β	Mediation effect SE	Mediation effect OR(95% CI)	Mediated proportion
Smoking Initiation	MV-Age	Sepsis	0.886(0.818-0.932)	-0.121	0.006	0.314(0.149-0.659)	-1.16	0.379	1.618(1.364-1.920)	0.481	0.14	0.046	1.15 (1.051-1.259)	29.106%
Smoking Initiation	TL	Sepsis	0.919(0.890-0.949)	-0.084	0.013	1.146(1.035, 1.270)	0.137	0.052	1.618(1.364-1.920)	0.481	-0.012	0.005	0.988 (0.978-0.998)	2.495%
BMI	MV-Age	Sepsis	0.906(0.895-0.916)	-0.099	0.004	0.314(0.149-0.659)	-1.16	0.379	1.511(1.375-1.661)	0.413	0.115	0.038	1.122 (1.041-,1.209)	27.845%
BMI	TL	Sepsis	0.956(0.937-0.976)	-0.045	0.01	1.146(1.035, 1.270)	0.137	0.052	1.511(1.375-1.661)	0.413	-0.006	0.003	0.994 (0.988-0.999)	14.528%
Smoking Initiation	MV-Age	COVID-19	0.886(0.818-0.932)	-0.121	0.006	2.289(1.343, 3.900)	0.828	0.272	2.289(1.342-3.900)	0.828	-0.1	0.062	0.940(0.801-0.999)	12.077%
Smoking Initiation	TL	COVID-19	0.919(0.890-0.949)	-0.084	0.013	1.101(1.016, 1.194)	0.096	0.041	2.289(1.342-3.900)	0.828	-0.001	0.004	0.998(0.997- 0.999)	1.208%
BMI	MV-Age	COVID-19	0.906(0.895-0.916)	-0.099	0.004	2.289(1.343, 3.900)	0.828	0.272	0.708(0.664-0.756)	-0.345	-0.082	0.027	0.921(0.874-0.971)	7.826%
BMI	TL	COVID-19	0.956(0.937-0.976)	-0.045	0.01	1.101(1.016, 1.194)	0.096	0.041	0.708(0.664-0.756)	-0.345	-0.004	0.002	0.996(0.992-0.999)	11.594%

The mediation effects were derived using the product-of-coefficients method, with A (exposure–mediator), B (mediator–outcome), and C (total exposure–outcome effect) expressed on a unified log-odds (β) scale. The indirect effect was calculated as A × B, and the proportion mediated as (A × B)/C. 'Total effect' indicates the effect of BMI or smoking initiation on sepsis, 'Direct effect A' implies the effect of smoking initiation or BMI on aging-related indices, 'Direct effect B' implies the effect of aging-related indices on sepsis and 'mediation effect' implies the effect of BMI or smoking initiation on sepsis through aging-related indices. The total effect, direct effect A, and direct effect B were derived using inverse-variance weighted (IVW). Body mass index (BMI); Telomere length (TL).

### 3.4 Immune Phenotypes Potentially Linking Biological Aging With COVID-19 and Sepsis

We presented a hypothesis that biological aging could lead to alterations in the levels of specific immunophenotyping, which in turn mediate infectious diseases. To explore this, we first performed MR analyses to assess the genetically predicted associations between biological aging traits and 731 immunophenotypes. We observed evidence that TL was significantly associated with 21 immunophenotypes (FDR-adjusted *P*<0.05). Next, we designed a mediation MR analysis to identify potential immunophenotypes that could act as mediators in the relationship between biological aging and infectious diseases. IVW MR analysis shows that TL/immunophenotypes pairs IgD^+^CD38^-^ B cell (OR, 1.319; 95% CI, 1.099 ∼ 1.582) in the these 21TL/immune trait pairs remained statistically significant with COVID-19 (FDR-adjusted p< 0.05, Table 4). Sensitivity analyses suggested that the identified immune factors were robust to pleiotropy (Supplementary Table 15-17). Finally, we quantified the mediating proportions of immune cells. The results of the IVW method of MR analysis showed that CD19 on IgD^+^CD38^-^ B cell immunocyte phenotypes mediated the association between TL with COVID-19 (18.75%) (Table 4). In contrast, the analyses of TL with sepsis, MV-Age with COVID-19, and MV-Age with Sepsis did not identify any immune phenotypes that met the mediation criteria, and no meaningful indirect effects were observed.

**Table 4:** Associations of immunophenotypes in the relationships between biological aging and COVID-19 and sepsis.

**Exposure**	**Mediator**	**Outcome**	**Direct effect (A)OR** **(95% CI)**	**Direct effect (A)β**	**Direct effect (A)SE**	**Direct effect (B)OR** **(95% CI)**	**Direct effect (B)β**	**Direct effect (B) SE**	**Total effect (C)OR** **(95% CI)**	**Total effect(C)β**	**Mediation effect β**	**Mediation effect SE**	**Mediation effect OR** **(95% CI)**	**Mediated proportion**
TL	CD19 on IgD+ CD38- B cell	COVID-19	1.319(1.099,1.582)	0.277	0.093	1.067(1.000,1.139)	0.065	0.033	1.101(1.016,1.194)	0.096	0.018	0.001	1.020(1.001, 1.040)	18.75%

The mediation effects were derived using the product-of-coefficients method, with A (exposure–mediator), B (mediator–outcome), and C (total exposure–outcome effect) expressed on a unified log-odds (β) scale. The indirect effect was calculated as A × B, and the proportion mediated as (A × B)/C

## 4 Discussion

In the present study, we conducted a comprehensive investigation into the potential association between multiple dimensional biological aging and incident of sepsis and COVID-19 using genetic instruments. We supported the hypothesis that the significance of aging-related healthy lifestyle interventions in providing direct insights into the biological aging to prevent the incidence of COVID-19 and sepsis in later life. Furthermore, leveraging large-scale genetic data, our analyses suggest that immunophenotypes may be involved in the associations between biological aging and susceptibility to COVID-19 and sepsis.

Life-threatening infectious conditions substantially increase the challenges in elderly demographics owing to our society's ongoing biological aging trajectory(Ljungström et al., 2019). Biological aging process entails a gradual accumulation of deteriorating biological functions over time, which ultimately amplifies the susceptibility and mortality of infections(Pei et al., 2022; Schumacher et al., 2021). The role of biological aging in the worsening of infectious symptoms has long been one of the hottest topics of today’s research(Jamie et al., 2021). As the multi-dimentional nature of human aging in health state, we used TL and MV-age to investigate the biological aging role in sepsis and COVID-19 outcomes. As generally accepted concept that aging is a multifactorial process, TL have been proposed a highly heritable and measurable “molecular signature” present throughout the life course(Allsopp et al., 1992; Childs et al., 2014; Codd et al., 2021; Vasa-Nicotera et al., 2005), we assessed the potential associations of TL on sepsis and COVID-19 using the MR approach. Our findings are consistent with those of a prospective observational study that illustrated a strong association between shortened leukocyte TL and increased susceptibility of critically (both sepsis and COVID-19) ill patients(Liu et al., 2020; Wang et al., 2020).

It is noteworthy that TL and MV-age indices represent different facets of biological aging, we further utilized the GWAS summary statistics of MV-age traits (including parental lifespan, health-span, extreme longevity, frailty, and DNA methylation-based epigenetic clocks) from the latest authoritative and appropriate consortia to augment the accuracy and precision of our effect estimates(Horvath et al., 2015; Revy et al., 2023; Rosoff et al., 2023). Consistent with our findings, recent MR studies on ageing have used epigenome-wide approaches to identify causal CpG sites that contribute to methylation-based epigenetic clocks(Ying et al., 2024). Whereas our work leverages genetically predicted ageing phenotypes to capture broader and more upstream biological processes that reflect systemic ageing. Taken together, these findings support the notion that biological aging measures, more closely related to health status than chronological age, serve as more reliable indicators of an individual’s biological age. Interestingly, longer genetically predicted telomere length increased the risk of sepsis but not COVID-19, whereas higher MV-aging scores showed the opposite pattern for sepsis but consistently increased COVID-19 risk. The discrepancy between TL and MV-Age effects on sepsis likely reflects the different biological processes. TL directly marks cellular aging, whereas MV-Age incorporates multiple aging factors, such as frailty and epigenetic changes, which may affect immune function more intricately(Rosoff et al., 2023). These complementary ageing domains may influence susceptibility to different infectious syndromes in different ways. Further exploration of their relationship, including both independent and combined effects on infection risk, is crucial to understanding their role in aging-related infection susceptibility(Han et al., 2025). These findings support the notion that biological aging signature, more closely related to health measures than chronological age, are seen as better biological age indicators. Therefore, we conducted a comprehensive analysis to explore the potential role of biological aging could be a potential risk target for the clinical incidence of sepsis and COVID-19 from multiple dimensions in older adults.

Recently, studies have sparked interest in healthy lifestyles, as they represent modifiable interventions to delay the pace of accelerating biological aging in life expectancy and age-related chronic disease(Fitzgerald et al., 2021; Gielen et al., 2018; Mamoshina et al., 2019). Identifying therapeutic interventions that promote 'healthy aging' and simultaneously the occurrence progression of multiple age-related pathological conditions is of paramount importance(Partridge et al., 2018). Our study extended previous studies and highlighted the high confidence associations of modifiable essential lifestyle factors (BMI and smoking) with different human aspects of biological aging indices with high levels of certainty(Fitzgerald et al., 2021; Klopack et al., 2022; Ni et al., 2023). Primary UVMR-analyses analyses provided additional support for the association between four modifiable lifestyle factors (cigarette smoking, inadequate physical activity, higher body weight, and abnormal metabolic index) and worsening outcomes in patients with sepsis. Interestingly, physical activity and metabolic index were shown to correlate with a higher incidence of complications and death during sepsis in the UVMR analysis, which did not persist after adjustment for other modifiable lifestyle factors (adiposity indicators and cigarette behaviors) in our multivariable analysis. As biological aging-related phenotype may influence the genetic risk of infectious disease. We tried to investigate the role of the aging-related modifiable lifestyles interventions on the occurrence and intensity of sepsis and COVID-19. To further explore the biological and clinical relevance of the observed associations, we examined several key aging-related lifestyle factors to assess their associations with the risk of infectious diseases. Our MVMR analysis highlighted the influence of BMI and smoking initiation persisted independent of other known modifiable lifestyle factors associated with sepsis-cause-specific mortality. Consistently, Genetically predicted body weight-related indicators such as BMI and smoking behaviors also showed significant associations with COVID-19 in MR analyses. Cigarette smoking is a well-established risk factor associated with a longer hospital stay and higher mortality in patients with severe sepsis or septic shock(Alroumi et al., 2018; Paulsen et al., 2017). A study conducted in Denmark encompassing a large cohort of 101,447 individuals revealed an intriguing association between higher BMI and an elevated risk of sepsis(Winter-Jensen et al., 2020). Our MR results are consistent with existing observational evidence, suggesting that aging-related modifiable lifestyle factors, such as body weight and smoking behaviors, are associated with susceptibility to severe infectious diseases. The differences in direction between sepsis and COVID-19 associations likely reflect disease-specific mechanisms or cohort characteristics. These findings highlight that MR results should be interpreted in the context of each specific infectious outcome.

To elucidate the potential biological pathway, we further employed mediated MR analysis to explore the potential mediating role of biological aging in the association between healthy lifestyles and the occurrence of infectious diseases. Genetically determined TL and MV-Age both played modest mediating roles in the total impact of BMI on the outcome of sepsis with individual mediation proportions of 1.586% and 31.476% respectively; these specific of biological aging traits (TL and MV-Age) mediated approximately 2.589% and 23.467% of the total effect of smoking behaviors on sepsis outcomes respectively. We further used hospitalized COVID-19 as the condition of severe infectious to verified the mediated role of biological aging between modifiable lifestyles and severe infectious diseases. We further observed that TL and MV-age may partially account for the associations between BMI and COVID-19, with estimated mediation proportions of 2.633% and 11.286%, respectively; similar patterns were observed for smoking behaviors, with proportions of 2.057% and 12.125%. Therefore, these MR analyses provide suggestive evidence that multi-dimensional biological aging traits may be involved in the associations between modifiable lifestyle factors and severe infectious diseases. Notably, the univariable MR suggested a negative total effect of smoking on sepsis, while the multivariable analysis indicated a positive ageing-adjusted effect. This difference may indicate that the univariable model captures pleiotropic genetic signals unrelated to ageing, whereas the multivariable model separates the ageing-related pathway through which smoking increases sepsis risk. Furthermore, colocalization analyses indicate that some genetic variants are shared between key exposures, such as BMI, MV-Age, smoking initiation, and outcomes such as COVID-19 and sepsis. This suggests that overlapping genetic architecture may partly underlie the associations observed in MR analyses. Shared genetic signals may influence both lifestyle traits and immune or inflammatory pathways, supporting a model in which genetic predisposition to smoking or higher BMI can affect immune function and infection risk. These findings highlight the importance of considering shared genetic mechanisms when interpreting lifestyle, disease relationships and may inform strategies to prevent infection in high-risk individuals. Therefore, Our MR results reinforce the notion that aging-related lifestyles, obesity and smoking behavior can be considered direct potential risk factors for increased susceptibility to the consequences of severe infectious diseases.

Widespread activation of immune cells of the systemic circulation is especially pronounced during aging, especially the corresponding immunoglobin protein levels in plasma highly correlated with COVID-19 (Egbert et al., 2021; Okeke et al., 2024; Schaum et al., 2020). Previous research has identified clinical and molecular factors linked to COVID-19 severity, and addressing biologically aging-related COVID-19 or sepsis requires understanding the factors that accelerate biological aging and increase associated risks. For example, a Mendelian randomization analysis showed that genetic liability to severe COVID-19 is associated with adverse outcomes, including immune and inflammation related pathways (Baranova et al., 2023). Additionally, circulating inflammatory proteins have been shown to confer both risk and protective effects on severe COVID-19 outcomes, implicating specific immunomodulatory factors in disease progression (Baranova et al., 2024). Undoubtedly, the immune system plays an essential role in promoting healthy aging, and experiences notable shifts in the composition of different the immune cell types in the bloodstream with advancing age (Santoro et al., 2021). In fact, when it comes to tracking health and lifestyle/health interventions, immunology aging signature may perform better than the well-established biological age measures(Ma et al., 2024; Terekhova et al., 2023). Our findings indicate that both TL and MV-Age exert only a small effect on sepsis, suggesting that their overall contribution to sepsis risk is limited. However, several adaptive immune cell subsets exhibited stronger mediation signals in the TL and COVID-19 pathway, suggesting that telomere-associated immune aging may influence infection risk through selective rather than generalized immunological biological pathway. In contrast, MV-Age, representing broader aging processes beyond immunity, may attenuate the specific mediation effects. Nevertheless, these results should be interpreted carefully, and larger studies with more comprehensive data are needed to further support our findings. A fundamental question is why do individuals manifest such wide differences in lifespan, health status across age, and susceptibility to COVID-19 and sepsis? One possibility is that variations in an immune trait contribute to these differences (Ahuja, 2023). We found signatures tracking biological aging-associated immunocompetence with COVID-19 and sepsis. Among the 731 immuno-phenotypes examined in the mediation analysis, B cell related traits showed the greatest number of significant associations with TL compared with other immune cell subsets. For example, CD19 on IgD^+^ CD38^-^ B cell immune traits showed suggestive mediation of the association between genetically predicted TL and COVID-19 risk, which may actively shape ageing phenotypes by accelerating telomere attrition, altering cytokine signaling, and increasing immunometabolic stress, thereby influencing susceptibility to infection.

Previous studies have shown that immune cell dysfunction, particularly impaired immune surveillance, is a key factor in increased infection susceptibility in older individuals(Liu et al., 2025). Telomere abnormalities are associated with an increase in senescent immune cells, which are less responsive and more prone to inflammation, potentially exacerbating infection severity. The increased production of pro-inflammatory cytokines and immune dysregulation during immune aging may be critical mechanisms driving COVID-19 susceptibility(Fu et al., 2025). These findings align with emerging studies implicating boosting immune system can offer a novel therapeutic opportunity to attenuate clinical deterioration in the elderly COVID-19 infected with SARS-CoV-2. However, the differences between the immune biological pathway for COVID-19 and sepsis may reflect disease-specific immune pathways, with distinct immune phenotypes playing a key role in sepsis and COVID-19 (Wang et al., 2025). Therefore, our study provides proof-of-concept evidence that the underlying immunocompetence for COVID-19 and sepsis is enriched in conditions that cause biological aging, offering a potential new therapeutic avenue for preventing or mitigating aging-related infectious diseases.

## 5 Conclusion and Limitation

In conclusion, our study provides genetically informed evidence linking biological aging, lifestyle-related traits and immune phenotypes with susceptibility to COVID-19 and sepsis. Our findings indicate that genetically informed risk stratification can facilitate earlier identification of individuals at heightened risk of age-related infection susceptibility. The implementation of modifiable healthy lifestyle interventions, beginning at midlife, appear to be crucial to reducing infectious disease burdens. Personalized, prevention-oriented lifestyle strategies may further help mitigate this aging-linked vulnerability. Notably, as the proportion of immunophenotype changes in elderly individuals, it is essential to emphasize the immune strategies to health biological aging for the management of infectious diseases.

Our study had some limitations which should be carefully interpreted. First, our analysis was based on GWASs conducted within populations of European ancestry, which may limit the generalizability of the findings to other populations. Although we applied multiple sensitivity tests, we cannot entirely dismiss the possibility of violation of the assumptions of independence and exclusion restrictions. It is important to acknowledge that the genetic component of biological aging represents only one aspect of the aging process. Future research could integrate broader dimensions of aging, like epigenetic aging indicators, longitudinal lifestyle and environmental factors, to provide a more comprehensive understanding of aging-related susceptibility(Argentieri et al., 2025). Although several immune cell subsets showed partial mediation of aging effects on COVID-19, no formal colocalization was performed due to limited power. These findings are considered suggestive and should be interpreted cautiously. In addition, sample overlap between lifestyle exposures (BMI, smoking, blood pressure) and sepsis outcomes may exist, as both are derived from the UK Biobank. Nevertheless, the instrumental variables were strong (F>10), and sensitivity analyses including MR-Egger, weighted median/mode, MR-PRESSO, and leave-one-out analyses produced consistent estimates, suggesting that any potential bias from overlap is likely minimal. Overall, the MR findings should be interpreted with caution.

## Ethics Approval and consent to participate

Not applicable.

## Clinical Trial

Not applicable

## Consent for publication

All participants provided written informed consent for the publication of anonymized data collected in this study.

## Funding

This work was supported by the National Natural Science Foundation of China (No. ZR2020MH138).

## CRediT authorship contribution statement

**Yuru Tang:** Writing – original draft, Visualization, Software, Resources, Methodology, Investigation, Formal analysis. **Xiao Gao:** Writing – review & editing, Validation, Methodology. **Huafang Ding:** Resources. **Lingli Kong:** Writing – review & editing, Conceptualization. **Xiaoyan Zhu:** Validation, Project administration, Conceptualization.

## Declaration of competing interest

The authors declare no conflict of interest.

## Data Availability

The data that has been used is confidential.

## Appendix Supplementary materials

Image, application 1

Image, application 2
