# Supplementary material for: Biological Aging, Immune Phenotypes, and Susceptibility to COVID-19 and Sepsis: A Mendelian Randomization Study
Source: Virus Res. 2026 Jun 2;370:199756. doi: 10.1016/j.virusres.2026.199756 (PMC13292545; doi:10.1016/j.virusres.2026.199756)
Supplement: Supplementary file 1 [file mmc1.doc]

**Supplementary Figures**

**Supplementary Methods**

**Inclusion criteria for candidate exposures and mediators**………………………………………………………2

**UVMR Analysis**…………………………………………………………………………………………………..…2

**Supplementary Figures of contents**

**Supplementary Figure 1-8. MR scatter plot, forest map, leave-one-out and funnel figures for the causal associations of health lifestyles with sepsis outcome under UVMR analysis**.................................................3-6

**Supplementary Figure 9-12. MR scatter plot, forest map, leave-one-out and funnel figures for the causal associations of positive lifestyles with each mediator (telomerase length and MV-Age) under UVMR analysis**.…….….....................................................................................................................................................7-8

**Supplementary Figure 13-14. MR scatter plot, forest map, leave-one-out and funnel figures for the causal associations of each mediator (telomerase length and MV-Age) with sepsis**.…….........................................….9

**Supplementary Figure 15-16 MR scatter plot, forest map, leave-one-out and funnel figures for the causal associations of modifiable aging-related lifestyles with COVID-19 outcome under UVMR analysis**................10

**Supplementary Figure 17-18. MR scatter plot, forest map, leave-one-out and funnel figures for the causal associations of each mediator (telomerase length or MV-Age) with COVID-19**..................................................11

**References** ..................................................................................................................................................................12

**Supplementary R Packages and MR Analytical Parameter Settings** .............................................................13-16

**Supplementary Methods**

1. **Inclusion criteria for candidate mediators**

Based on literature reviews of observational and MR studies, we selected 30 candidate exposures (8 lifestyle factors) and biological aging-related mediator. First, based on collective scientific knowledge, mediator longevity might lie on the pathways from 8 lifestyle to infectious disease outcome. Second, modifiable through lifestyle behavior improvements or accessible clinical interventions may potentially sepsis outcome by mediating human biological aging. Third, human biological aging as candidate mediators were relatively prevalent and had a significant public health implication on sepsis outcome. Fourth, reliable genome-wide association studies for multi- ageing traits were available^1,2^. Considering the substantial death burden caused by diseases, the close correlation between modifiable lifestyles factors and diseases, and the extensive attention of the international community, we focused on associated with biological aging and infectious disease mortality outcome.

1. **UVMR Analysis**

To investigate the causal associations between health lifestyles and sepsis outcomes under UVMR (Univariable Mendelian Randomization) analysis, several visualizations were generated.

- 1. **MR Scatter Plot**

The scatter plot was used to illustrate the relationship between instrumental variables (IVs) and the outcomes. The plot was generated by plotting the effect estimates of each IV on the outcome along with their respective confidence intervals.

**2.2 Forest Plot**

The forest plot displays the effect estimates for each individual study, along with the associated 95% confidence intervals. This figure helps to summarize the individual study effects and provides an overall assessment of the association.

**2.3 Leave-One-Out Analysis**

A leave-one-out analysis was conducted to check the robustness of the causal associations. This method systematically excluded one study at a time to evaluate whether the results were sensitive to any specific study.

**2.4 Funnel Plot**

The funnel plot was used to assess publication bias by plotting the standard errors against the effect sizes. A symmetrical funnel suggests the absence of bias.

**Supplementary Figure 1. Scatter plot (A), forest plot (B), and “leave-one-out” analysis (C) and funnel plot (D) for MR causal associations of smoking initiation and sepsis outcome under UVMR analysis**

**Supplementary Figure 2. Scatter plot (A), forest plot (B), and “leave-one-out” analysis (C) and funnel plot (D) for the causal associations of cigarettes-per-day and sepsis outcome under UVMR analysis**

**Supplementary Figure 3. Scatter plot (A), forest plot (B), and “leave-one-out” analysis (C) and funnel plot (D) for the causal associations of the never status of smoking sepsis outcome under UVMR analysis**

**Supplementary Figure 4. Scatter plot (A), forest plot (B), and “leave-one-out” analysis (C) and funnel plot (D) for the causal associations of leisure screen time and sepsis outcome under UVMR analysis**

**Supplementary Figure 5. Scatter plot (A), forest plot (B), and “leave-one-out” analysis (C) and funnel plot (D) for the causal associations of HDL gcholesterol and sepsis outcome under UVMR analysis**

**Supplementary Figure6. Scatter plot (A), forest plot (B), and “leave-one-out” analysis (C) and funnel plot (D) for the causal associations of waist circumferences (WC) and sepsis outcome under UVMR analysis**

**Supplementary Figure 7. Scatter plot (A), forest plot (B), and “leave-one-out” analysis (C) and funnel plot (D) for the causal associations of body mass index (BMI) and sepsis outcome under UVMR analysis**

**Supplementary Figure 8. Scatter plot (A), forest plot (B), and “leave-one-out” analysis (C) and funnel plot (D) for the causal associations of body fat percentage (BFP) and sepsis outcome under UVMR analysis**

**Supplementary Figure 9. MR scatter plot, forest map, leave-one-out and funnel figures for the causal associations of BMI with telomerase length under UVMR analysis**

**Supplementary Figure 10. MR scatter plot, forest map, leave-one-out and funnel figures for the causal associations of BMI with MV-age under UVMR analysis**

**Supplementary Figure 11. MR scatter plot, forest map, leave-one-out and funnel figures for the causal associations of smoking initiation with telomerase length under UVMR analysis**

**Supplementary Figure 12. MR scatter plot, forest map, leave-one-out and funnel figures for the causal associations of smoking initiation with MV-age under UVMR analysis**

**Supplementary Figure 13. MR scatter plot, forest map, leave-one-out and funnel figures for the causal associations of telomerase length with sepsis outcome under UVMR analysis**

**Supplementary Figure 14. MR scatter plot, forest map, leave-one-out and funnel figures for the causal associations of MV-age with sepsis outcome under UVMR analysis**

**Supplementary Figure 15. MR scatter plot, forest map, leave-one-out and funnel figures for the causal associations of BMI with COVID-19 outcome under UVMR analysis**

**Supplementary Figure 16. MR scatter plot, forest map, leave-one-out and funnel figures for the causal associations of smoking initiation with COVID-19 outcome under UVMR analysis**

**Supplementary Figure 17. MR scatter plot, forest map, leave-one-out and funnel figures for the causal associations of telomerase length with COVID-19**

**Supplementary Figure 18. MR scatter plot, forest map, leave-one-out and funnel figures for the causal associations of MV-age with COVID-19**

**References**

1. Codd V., Wang Q., Allara E., Musicha C., Kaptoge S., Stoma S., et al. Polygenic basis and biomedical consequences of telomere length variation. *Nat Genet* 2021;53(10):1425–33. Doi: 10.1038/s41588-021-00944-6.

2. Rosoff DB., Mavromatis LA., Bell AS., Wagner J., Jung J., Marioni RE., et al. Multivariate genome-wide analysis of aging-related traits identifies novel loci and new drug targets for healthy aging. *Nat Aging* 2023;3(8):1020–35. Doi: 10.1038/s43587-023-00455-5.

**Supplementary R Packages and MR Analytical Parameter Settings**

rm(list = ls())

Sys.setenv(OPENGWAS_JWT="")

PATH<-''

setwd(PATH)

library(ggplot2)

library(plyr)

library(dplyr)

library(tidyr)

library(data.table)

library(TwoSampleMR)

library(ieugwasr)

library(MendelianRandomization)

library(ieugwasr)

TL<-extract_instruments(outcomes ="", p1=5e-08,clump =T,r2=0.001,kb=10000, p2=5e-08)

head(TL)

TL$id.exposure<-"Telomerase length"

TL$exposure<-"Telomerase length"

head(TL)

sepsis<-extract_outcome_data(snps=TL$SNP,outcomes="",proxies=T,maf_threshold = 0.01)

head(sepsis)

sepsis$id.outcome<-"sepsis"

sepsis$outcome<-"sepsis"

sepsis_ver<-subset(sepsis,pval.outcome>5e-8)

data_h<-harmonise_data(exposure_dat=TL,outcome_dat=sepsis,action=2)

head(data_h)

mr_outcome<-mr(data_h)

mr_outcome

mr_outcome$OR<-exp(mr_outcome$b)

mr_outcome$LowerCI<-exp(mr_outcome$b-1.96*mr_outcome$se)

mr_outcome$UowerCI<-exp(mr_outcome$b+1.96*mr_outcome$se)

mr_outcome

write.csv(mr_outcome[1:5,1:12],"mr_outcome.csv",quote = F,row.names = F)

p1 <- mr_scatter_plot(mr_outcome, data_h)

p1[[1]]

#Leave-one-out plot

mr_outcome_loo <- mr_leaveoneout(data_h)

p3 <- mr_leaveoneout_plot(mr_outcome_loo)

p3[[1]]

#Forest plot

mr_outcome_single <- mr_singlesnp(data_h)

p2 <- mr_forest_plot(mr_outcome_single)

p2[[1]]

mr_outcome_single <- mr_singlesnp(data_h)

p4 <- mr_funnel_plot(mr_outcome_single)

p4[[1]]

library(ggpubr)

ggarrange(p1[[1]],p2[[1]],p3[[1]],p4[[1]],labels=c("A","B","C","D"),nrow=2,ncol = 2)

#heterogeneity

H<-mr_heterogeneity(data_h)

H

#pleiotropy_test

ple <- mr_pleiotropy_test(data_h)

ple

data_h$samplesize.exposure=as.numeric(data_h$samplesize.exposure)

data_h$samplesize.outcome=as.numeric(data_h$samplesize.outcome)

head(data_h)

directionality_test(data_h)

mydata<-data_h

mrinput <- mr_input(bx =mydata$beta.exposure, bxse = mydata$se.exposure,

by = mydata$beta.outcome, byse= mydata$se.outcome,

correlation =matrix(),

exposure = "exposure", outcome = "outcome")

radial_model<-RadialMR::ivw_radial(r_input =mrinput, alpha = 0.05,

weights = 1, tol = 0.0001, summary = TRUE)

RadialMR::plot_radial(radial_model)

data_radial<-radial_model$data

View(data_radial)

head(data_radial)

#write data_h

write.csv(data_radial,"data_radial.csv",quote = F,row.names = F)

# delete outlier

data_radial2 <- subset(data_radial,Outliers=="Outlier")

#View(data_radial2)

head(data_radial2)

a<-row.names(data_radial2)

a

b<-as.numeric(a)

b

data_h<-data_h[-b,]

write.csv(data_h,"data_h-2.csv",quote = F,row.names = F)

mr_outcome<-mr(data_h)

mr_outcome

mr_outcome$OR<-exp(mr_outcome$b)

mr_outcome$LowerCI<-exp(mr_outcome$b-1.96*mr_outcome$se)

mr_outcome$UowerCI<-exp(mr_outcome$b+1.96*mr_outcome$se)

mr_outcome

write.csv(mr_outcome[1:5,1:12],"mr_outcome.csv",quote = F,row.names = F)

#heterogeneity

H<-mr_heterogeneity(data_h)

H

#pleiotropy test

ple <- mr_pleiotropy_test(data_h)

ple

library(MRPRESSO)

mr_presso(BetaOutcome = "beta.outcome",BetaExposure="beta.exposure",

SdOutcome = "se.outcome", OUTLIERtest = T, DISTORTIONtest = T,SdExposure = "se.exposure",data=data_h)

mr_presso

data_h$R2<-data_h$beta.exposure*data_h$beta.exposure*2*(data_h$eaf.exposure)*(1-data_h$eaf.exposure)

data_h$F<-(data_h$samplesize.exposure-2)*data_h$R2/(1-data_h$R2)
